# Population-based assessment of nusinersen efficacy in children with spinal muscular atrophy: a 3-year follow-up study

**DOI:** 10.1093/braincomms/fcac269

**Published:** 2022-10-31

**Authors:** Féline E V Scheijmans, Inge Cuppen, Ruben P A van Eijk, Camiel A Wijngaarde, Marja A G C Schoenmakers, Danny R van der Woude, Bart Bartels, Esther S Veldhoen, Irene L B Oude Lansink, Ewout J N Groen, Fay-Lynn Asselman, Renske I Wadman, W Ludo van der Pol

**Affiliations:** Department of Neurology, UMC Utrecht Brain Center, University Medical Center Utrecht, 3584 CX, Utrecht, The Netherlands; Department of Neurology, UMC Utrecht Brain Center, University Medical Center Utrecht, 3584 CX, Utrecht, The Netherlands; Department of Neurology, UMC Utrecht Brain Center, University Medical Center Utrecht, 3584 CX, Utrecht, The Netherlands; Biostatistics and Research Support, Julius Centre for Health Sciences and Primary Care, University Medical Centre Utrecht, 3584 CX, Utrecht, The Netherlands; Department of Neurology, UMC Utrecht Brain Center, University Medical Center Utrecht, 3584 CX, Utrecht, The Netherlands; Child Development and Exercise Center, Wilhelmina Children’s Hospital, University Medical Center Utrecht, 3584 CX, Utrecht, The Netherlands; Child Development and Exercise Center, Wilhelmina Children’s Hospital, University Medical Center Utrecht, 3584 CX, Utrecht, The Netherlands; Child Development and Exercise Center, Wilhelmina Children’s Hospital, University Medical Center Utrecht, 3584 CX, Utrecht, The Netherlands; Pediatric Intensive Care Unit, Wilhelmina Children’s Hospital, 3584 EA, Utrecht, The Netherlands; Department of Rehabilitation, Physical Therapy Science and Sports, UMC Utrecht Brain Center, University Medical Center Utrecht, 3584 CX, Utrecht, The Netherlands; Department of Neurology, UMC Utrecht Brain Center, University Medical Center Utrecht, 3584 CX, Utrecht, The Netherlands; Department of Neurology, UMC Utrecht Brain Center, University Medical Center Utrecht, 3584 CX, Utrecht, The Netherlands; Department of Neurology, UMC Utrecht Brain Center, University Medical Center Utrecht, 3584 CX, Utrecht, The Netherlands; Department of Neurology, UMC Utrecht Brain Center, University Medical Center Utrecht, 3584 CX, Utrecht, The Netherlands

**Keywords:** spinal muscular atrophy, nusinersen, treatment, motor function, children

## Abstract

Nusinersen (Spinraza®) improves survival of infants with hereditary proximal spinal muscular atrophy and motor function in children up to 12 years. Population-based assessments of treatment efficacy are limited and confined to select cohorts of patients. We performed a nationwide, population-based, single-centre cohort study in children with spinal muscular atrophy younger than 9.5 years at start of treatment in line with reimbursement criteria in the Netherlands. We assessed age-relevant motor function scores, the need for tube feeding, hours of ventilatory support and documented adverse events. We used linear mixed modelling to assess treatment effects. We compared motor function during treatment with natural history data and to individual trajectories of muscle strength and motor function before the start of treatment. We included 71 out of 72 Dutch children who were treated (median age 54 months; range 0–117) and followed them for a median of 38 months (range 5–52). We observed improvement of motor function in 72% and stabilization in another 18% of the symptomatic children, which differed from the natural disease course in a matched cohort of which we had previously collected natural history data. Longitudinal analysis showed that motor function improved up to a median of 24 months (range 12–30) of treatment after which it stabilized. Shorter disease duration at start of treatment resulted in better treatment efficacy (*P* < 0.01). Sixteen children (23%) achieved new motor milestones. Bulbar and respiratory function did not improve significantly during treatment. In 15 patients from whom treatment-naïve data were available, the pre-treatment trajectory of motor function decline changed to stabilization or improvement after the start of treatment. We documented 82 adverse events after 934 injections (9%) in 45 patients. None of the adverse events led to treatment discontinuation. Intrathecal nusinersen treatment is safe and improves or stabilizes motor function in 90% of young children with spinal muscular atrophy types 1c–3a. We did not observe improvement of respiratory and bulbar functions.

## Teaser

Scheijmans *et al*. describe real-world efficacy and safety of treatment of children with hereditary proximal spinal muscular atrophy (5q-SMA) with the antisense oligonucleotide nusinersen. Motor function initially improved during treatment, but reached a plateau phase after a median treatment duration of 24 months. Shorter disease duration was associated with higher likelihood of motor function improvement.

## Graphical Abstract

**Graphical abstract:**
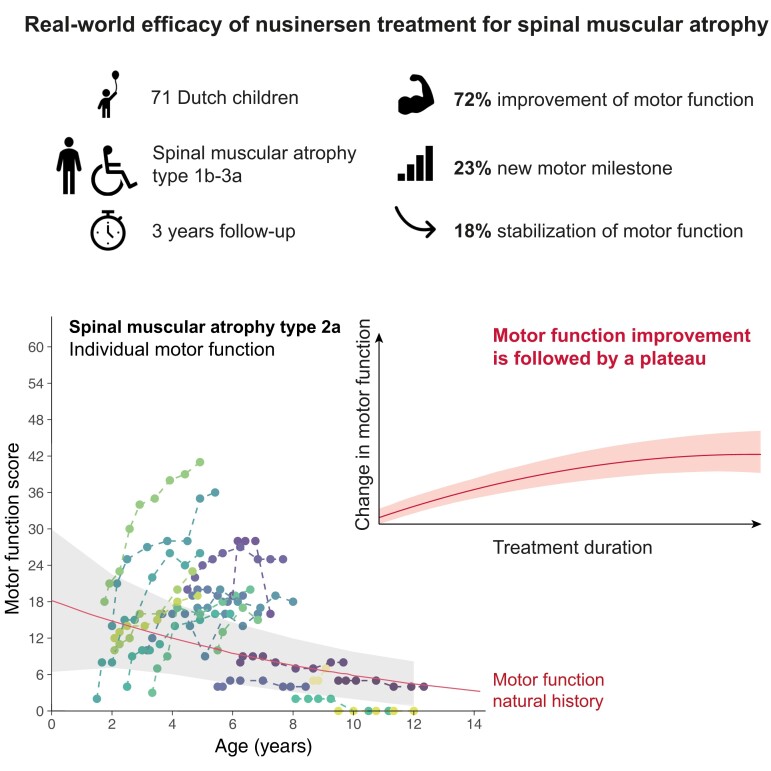

## Introduction

Hereditary proximal spinal muscular atrophy (SMA) is caused by deficiency of survival motor neuron (SMN) protein due to loss of the *SMN1* gene function.^1^ Its natural history is characterized by deteriorating muscle strength and motor function throughout life.^2–6^ SMA has a striking variability in disease severity. Ranging from an early infantile-onset form with severely impaired motor development (type 1), childhood-onset forms type 2 and type 3 with delayed gross motor development and progressive loss of motor function, to an adult-onset form with generally limited disability (type 4).^7^ Variability in disease severity between patients is partly explained by the inverse correlation with the *SMN2* copy number.^3,8,9^

Nusinersen (Spinraza®) is an antisense oligonucleotide that modifies *SMN2* splicing, thereby increasing SMN protein production. It is the first approved drug for the treatment of patients with SMA. Clinical trials showed that nusinersen improves survival of infants with SMA type 1 and motor function of infants and children with SMA types 1 and 2.^10,11^ After the regulatory approval of nusinersen in the USA (2016) and Europe (2017), an increasing number of observational studies and one meta-analysis documented beneficial effects on motor function in adolescents and adult patients with SMA types 2 and 3.^12–24^ Population-based, real-world data from larger cohorts of children with SMA would allow evaluation of the real-world efficacy of nusinersen but are still scarce.^4–6^ We here present data on motor, respiratory and bulbar function, and adverse events of 98% of the Dutch children with SMA types 1, 2 and 3a, eligible for reimbursement, who started treatment with nusinersen before July 2019.

## Materials and methods

### Patients

Treatment of SMA is centralized in the Netherlands at the SMA center in the University Medical Center Utrecht (UMCU). We therefore enrolled all patients with SMA who fulfilled the Dutch reimbursement criteria (i.e. patients younger than 9.5 years at start of treatment and 2 or 3 *SMN2* copies) between May 2017 and July 2019. All patients in this cohort had previously been included in an ongoing prospective population-based prevalence cohort study in the Netherlands and had agreed to have their clinical data included in the national SMA registry.^2,3,5^

We used historical natural history data from the Dutch SMA Registry.^2,3,5^ All patients in this natural history cohort were treatment naive. Our natural history study started in September 2010 and it now contains data of the very large majority of patients with SMA who live in the Netherlands. Follow-up of included patients continues until today. Motor function of all included patients was assessed at inclusion (baseline) and during follow-up every one to 5 years. For this study, we matched patients from the natural history cohort based on SMA type and age and included only patients for which at least two longitudinal treatment naive assessments were available.

We used age at onset and acquired motor skills to define SMA types 1–3 according to the SMA classification.^3,5,7^ In case of discrepancy between age at onset and reached motor milestones, the latter determined the final diagnosis. SMA type 1 (a, b and c) was defined by an onset before 6 months and the inability to sit independently at any time. Patients with type 1b show signs of hypotonia after the neonatal period and will never have head control or will ever be able to roll. Type 1c includes patients who meet the criteria of type 1, and not type 2, but show a relative better performance in motor skills, including head control or rolling from supine to prone, or at least to one side at any stage in life. Patients with SMA type 2 had onset between the age of six and 18 months and learned to sit (type 2a) or even stand but not walk assisted (type 2b). Patients with SMA type 3 developed weakness after the age of 18 months and learned to walk independently. Patients with type 3 were further divided into SMA type 3a (disease onset before the age of 3 years old) and type 3b (disease onset after the age of 3 years old).

We used multiplex ligation-dependent probe amplification (SALSA MLPA kit P021–B1–01 (MRC-Holland) to confirm the homozygous deletion of the *SMN1* gene and to determine *SMN2* copy number in all patients.

### Assessments of motor function, respiratory function and bulbar function

Three pediatric physical therapists (MAGCS, DW and BB) assessed motor function in all patients using the Children's Hospital of Philadelphia Infant Test of Neuromuscular Disorders (CHOP-INTEND)^27,28^ and Hammersmith Infant Neurological Examination-2 (HINE-2) for patients with SMA type 1,^29^ and Hammersmith Functional Motor Scale Expanded (HFMSE)^30,31^ for patients with SMA types 2 and 3. We used WHO definitions for acquired motor milestones.^32^ We assessed motor function prior to treatment and after three, four and five injections. After the fifth injection, we repeated assessments with every injection in presymptomatic children and those with SMA type 1. Children with SMA types 2 and 3 were assessed every 6 months. Patients were usually assessed at the day of intrathecal injection, with a maximum interval of 2 weeks.

We documented respiratory (i.e. hours of (non-)invasive ventilation) and bulbar functions (i.e. need for tube feeding) at least every 6–12 months.

Adverse events were systematically assessed using a questionnaire at every visit for nusinersen administration as well as during the yearly outpatient follow-up visits.

### Treatment

Treatment with nusinersen was available from May 2017 for children with SMA type 1 (as part of an expanded access program) and from January 2018 for children up to 6 years of age with SMA types 2 and 3 (preliminary reimbursement arrangement). The final reimbursement arrangement included children with SMA younger than 9.5 years, including presymptomatic children, with 2 or 3 *SMN2* copies came into effect in August 2018.^33^ Exclusion criteria for nusinersen treatment were as prescribed by the manufacturer.^34^ We applied lidocaine/prilocaine cream prior to lumbar intrathecal administration of nusinersen in patients less than 1-year old and general anaesthesia with inhalation anaesthetics or intravenously administered propofol at the intensive care unit or operation theatre for children of 1 year and older. No children required consistent angiography or CT guidance for their lumbar punction. Treatment as per protocol started with a loading dose at days zero, 14, 28 and 63 followed by intrathecal injections every 4 months.^34^

We defined treatment response as an improved disease trajectory in comparison to the known natural history patterns, defined by slow but continuous deterioration of motor function.^2,4–6^ Response to treatment therefore included both improvements of motor function and stabilization, i.e. remaining within the day-to-day variation of specific motor function scales (e.g. 4 points on the CHOP-INTEND scale, 2 points on the HINE-2 scale for type 1, 2 HFMSE points for types 2 and 3). With no progressive decline on two consecutive assessments.^2,5,10,11^

### Ethical approval

This cohort is part of an ongoing study. The local Medical Ethical Committee approved the study protocol, which is registered at the Dutch registry for clinical studies and trials (NL29692.041.09). We obtained written and oral informed consent of both parents or legal guardians of each patient.

### Statistical analyses

We used descriptive statistics to describe baseline characteristics. Continuous variables were presented as median (range), and categorical variables as frequency (percentage). We used linear mixed models (LMM) to estimate the population-average change in motor function from baseline to 36 months of treatment for the CHOP-INTEND, HINE-2 (SMA type 1) and up to 30 months of treatment for the HFMSE total score (SMA types 2 and 3). The fixed part of the model contained the baseline score and time since first injection in months. A random intercept and slope for time since first injection per patient were added as random effects. We evaluated the effect of time both as linear and as non-linear (quadratic) pattern. Likelihood ratio tests were used to determine significance. Bootstrapping (*n* = 1000) was used to estimate 95% confidence intervals.

We performed separate LMM to analyse response to treatment based on disease duration before treatment for SMA types 1, 2 and 3. We dichotomized patients on the median disease duration before treatment and added the interaction between disease duration and time since first injection. We used R software (R-3.5.1 for Windows with RStudio version 1.1.456, R Foundation for Statistical Computing, Vienna, Austria) for all statistical analyses. Significance level was set at *P* < 0.05.

### Data availability

The presented model summary statistics allow full reproduction of all LMMs. Additional data supporting our findings are available upon reasonable request.

## Results

Eighty-one patients were screened for eligibility of nusinersen treatment. Ten children were excluded from this study, due to the following reasons: palliative care was initiated in five infants (6%) with SMA type 1b who were already severely affected at presentation, three patients (4%) were excluded due to clinical trial participation elsewhere (one with nusinersen, one with onasemnogene abeparvovec-xioi and one with risdiplam), in one patient (1%) with SMA type 1c intrathecal injections were technically impossible due to complete thoracolumbar fusion after scoliosis surgery, and finally the parents of one child (1%) did not give informed consent for participation.

Patient characteristics of symptomatic children (*n* = 69) and their baseline values of motor, respiratory and bulbar function are presented in Table 1.

**Table 1:** Baseline characteristics of symptomatic patients treated with nusinersen

	All patients (*n* = 69)	SMA type 1 (*n* = 23)	SMA type 2 (*n* = 30)	SMA type 3 (*n* = 16)
SMA type ***n*** (%)				
A		0 (0)	20 (67)	16(100)
B		5 (25)	10 (33)	0 (0)
C		18 (75)	n.a	n.a.
Male sex ***n*** (%)	42 (60.9)	18 (78)	15 (50)	9 (56)
Age at disease onset in months	9 (0–36)	4.5 (0–13)*	10.3 (0–20)	19 (12–36)
Age at diagnosis in months	16 (0–82)	8 (2–22)	18 (0–36)	36 (0–82)
Disease duration at start of therapy	43 (0–114)	28 (0.5–113)	48 (5–114)	46.5 (6–95)
Age at first dose in months	54 (2–117)	37 (2–117)	55.5 (18–115)	68 (39–113)
*SMN2* copy number ***n*** (%)				
2	7 (10)	6 (26)	0 (0)	1** (6)
3	52 (75)	17 (74)	30 (100)	5 (31)
4	10 (15)	0 (0)	0 (0)	10 (62)
CHOP-INTEND score	31 (6–46)	31 (6–46)	n.a.	n.a.
HINE-2 score	0 (0–7)	0 (0–7)	n.a.	n.a.
HFMSE score	19 (0–62)	n.a.	11 (0–38)	47.5 (34–62)
WHO motor milestone score	1 (0–6)	0 (0)	1 (0–4)	6 (2–6)
None	24 (40)	23 (100)	1 (3)	0 (0)
Sitting without support	20 (31)	0 (0)	16 (53)	0 (0)
Standing with assistance	5 (7)	0 (0)	4 (13)	3 (19)
Hands and knees crawling	0 (0)	0 (0)	4 (13)	0 (0)
Walking with assistance	1 (1)	0 (0)	3 (10)	0 (0)
Standing alone	1 (1)	0 (0)	2 (7)	1(6)
Walking alone	12 (18)	0 (0)	0 (0)	12 (75)
Use of ventilator support ***n*** (%)				
None	53 (77)	11 (48)	26 (87)	16 (100)
Non-invasive ventilation	13 (19)	9 (39)	4 (13)	0 (0)
Hours per day of non-invasive ventilation	12 (8–16)	13 (8–16	10.5 (10–12)	n.a.
Invasive ventilation	4 (6)	4 (17)	0 (0)	0 (0)
Hours per day of invasive ventilation	22.5 (12–24)	22.5 (12–24)	n.a.	n.a.
Dependent on feeding tube ***n*** (%)	24 (35)	16 (30)	9 (30)	0 (0)
Nasogastric tube	6 (8)	5 (22)	2 (7)	0 (0)
Gastrostomy	18 (26)	11 (48)	7 (23)	0 (0)

SMA = spinal muscular atrophy; *n* = number; n.a. = not applicable; *SMN2* = survival motor neuron 2 gene; CHOP-INTEND = Children’s Hospital of Philadelphia Infant Test of Neuromuscular Disorders; HINE-2 = Hammersmith Infant Neurological Examination 2; WHO = World Health Organization; HFMSE = Hammersmith Functional Motor Scale Expanded.

All outcomes are given in median (range) unless otherwise stated.

* = Age at onset was between 8 and 13 months in four infants with SMA type 1c. Age at onset was particularly late in one infant (13 months) due to delay of symptom recognition.

** = One patient with confirmation of two *SMN2* copies and c.859G > C mutation in exon 7 of *SMN2.*

Five children (1 with type 1c, 3 with type 2a and 1 with type 2b) ended treatment with nusinersen during follow-up and switched to oral risdiplam as part of the JEWELFISH study (NCT03032172). One child (type 1c) died due to a non-treatment related event after 10 injections. All available follow-up data from these six children are included in our analyses.

### Survival, motor function and milestones in SMA type 1

We included 23 patients with SMA type 1 (5 with type 1b and 18 with type 1c). Nine patients (39%) learned to sit after 3–13 injections (Table 2), of whom 4 had SMA type 1b (baseline CHOP INTEND range 17–46) and 5 type 1c (baseline CHOP-INTEND range 29–42). One patient with type 1c lost the ability to sit due to scoliosis progression.

**Table 2:** Clinical outcome measures in patients with SMA type 1 treated with nusinersen

	SMA type 1b (*n* = 5)	SMA type 1c (*n* = 18)
Follow-up in months	41 (29–46)	45.5 (19–52)
Number of injections	13 (10–15)	14 (7–16)
		
CHOP-INTEND score	56 (23–58)	40 (15–54)
△CHOP-INTEND score	25 (12–39)	8 (1–18)
HINE-2 score	16 (15–19)	4 (0–9)
△HINE-2 score	15.5 (12–19)	3.5 (0–9)
WHO motor milestone score	1 (0–1)	0 (0–1)
Use of ventilator support ***n*** (%)		
None	3 (60)	4 (22)
Non-invasive ventilation	2 (40)	10 (56)
Hours of non-invasive ventilation	13.5 (13–14)	12 (11–13)
Invasive ventilation	0 (0)	4 (22)
Hours of invasive ventilation	n.a.	12 (12–24)
		
Dependent on feeding tube ***n*** (%)		
None	1 (20)^a^	4 (22)
Nasogastric tube	0 (0)	0 (0)
Gastrostomy	4 (80)	9 (50)

SMA = spinal muscular atrophy; *n* = number; CHOP-INTEND = Children’s Hospital of Philadelphia Infant Test of Neuromuscular Disorders; △CHOP-INTEND = difference in CHOP-INTEND score between baseline and last follow-up assessment; HINE-2= Hammersmith Infant Neurological Examination-2; △HINE-2 = difference in HINE-2 score between baseline and last follow-up assessment; WHO = World Health Organization.

Outcomes present the data of last follow-up assessment, unless otherwise stated. All outcomes are given in median (range) unless otherwise stated.

^a^ Swallow examination shows silent aspiration, parents refused feeding tube.

**Figure 1:**
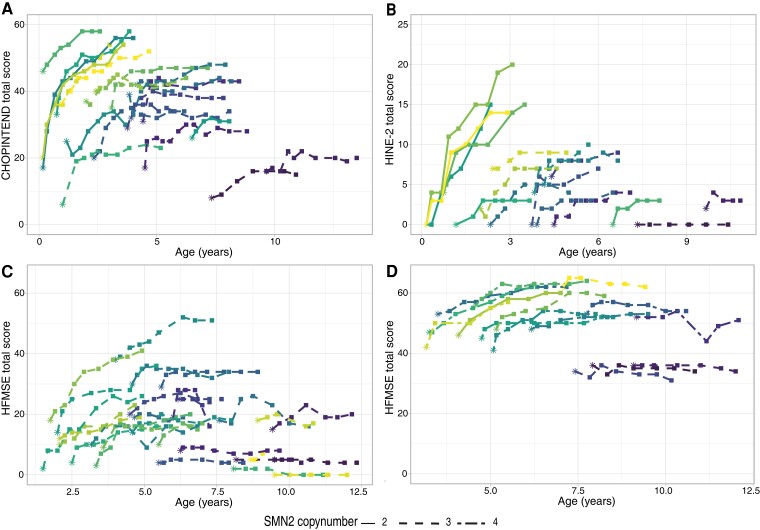
**CHOP-INTEND, HINE-2 and HFMSE scores of individual patients during treatment with nusinersen.** Motor function trajectories reflected by CHOP-INTEND, HINE-2 and HFMSE scores in symptomatic children with SMA types 1b-3a. Each individual (coloured) line represents one patient. Baseline assessment is indicated by asterisks, while follow-up assessments are indicated by squares. Effects of nusinersen treatment were associated with disease duration at treatment initiation and age at the start of treatment (likelihood ratio test: *P* < 0.001). (**A**) CHOP-INTEND scores in patients with SMA type 1, including types 1b and 1c. (**B**) HINE-2 scores in patients with SMA type 1, including types 1b and 1c. (**C**) HMFSE scores in patients with SMA type 2, including types 2a and 2b. (**D**) HFMSE scores in patients with SMA type 3a.

**Figure 2:**
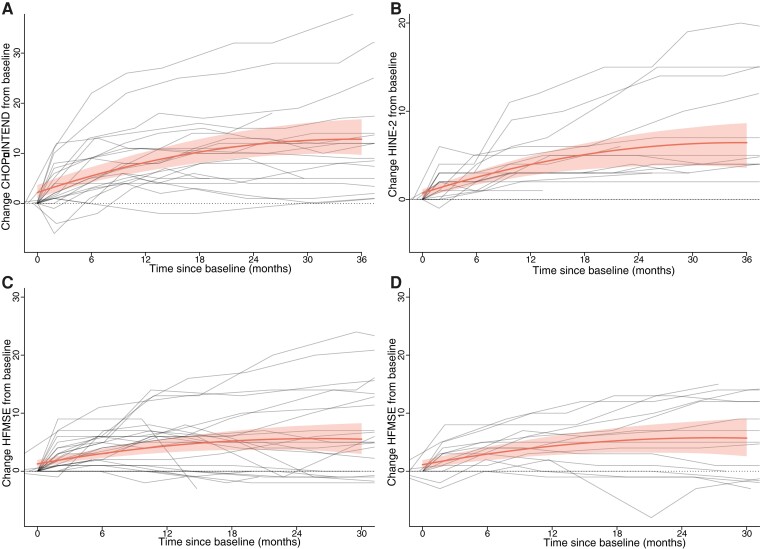
**Linear mixed model trajectory of motor function in patients with SMA types 1, 2 and 3 treated with nusinersen.** Motor function trajectories analysed with a linear mixed model with repeated measures, including bootstrapping (*n* = 1000) to estimate 95% confidence intervals. The grey lines represent individual trajectories. The red line and grey area reflect the mean changes in motor function over time with confidence interval, respectively. (**A**) Change from baseline in CHOP-INTEND score in all patients with SMA type 1 during treatment. (**B**) Change from baseline in HINE-2 score in patients with SMA type 1 during treatment. (**C**) Change from baseline in HFMSE score in patients with SMA type 2 during treatment. (**D**) Change from baseline in HFMSE score in patients with SMA type 3a during treatment.

CHOP-INTEND scores for 21 patients with SMA types 1b and 1c are shown in Fig. 1A. Eighteen children (85%) showed an improvement of ≥4 points on the CHOP-INTEND scale. CHOP-INTEND increments varied, were not linear (*P* < 0.001) and depended significantly on disease duration before start (*P* < 0.001). There was a mean increase in CHOP-INTEND scores from baseline of 3.3 (CI 2.7–4.0) points after 6 months of treatment, 6.0 (CI 4.8–7.1) points after 12 months, 8.0 (CI 6.3–9.7) points after 18 months, 9.6 (CI 7.5–11.6) points after 24 months, 10.4 (CI 7.8–12.9) points after 30 months and 10.6 (CI 7.5–13.7) after 36 months of treatment, all in comparison to baseline scores (Fig. 2A). After 30 months of treatment, group analysis showed a plateau of motor function gains.

HINE-2 scores of 17 patients did not deteriorate during follow-up and are shown in Fig. 1B. Increases in HINE-2 scores were non-linear in the first 18 months, i.e. a mean increase of 1.8 (CI 1.3–2.2) points after 6 months of treatment, 3.2 (CI 2.3–4.1) points after 12 months, 4.4 (CI 2.9–5.7) points after 18 months, 5.1 (CI 3.4–6.8) points after 24 months, 5.6 (CI 3.5–7.8) points after 30 months and 5.7 (CI3.1–8.3) points after 36 months of treatment (Fig. 2B, Table 2). Shorter disease duration at treatment initiation was associated with better treatment response (*P* < 0.001). None of the children showed a decline of motor function during treatment.

### Motor function assessments and milestones in SMA type 2

We included 30 patients with SMA type 2 (20 with type 2a and 10 with type 2b). Four children with SMA type 2a regained the ability to sit independently (after three to seven injections), one child with type 2a gained the ability to walk with assistance (after nine injections). Two children with SMA type 2b learned to stand without support (after four and seven injections, respectively) (Table 3). None of the children lost these new milestones during follow-up.

**Table 3:** Clinical outcome measures in patients with SMA types 2 and 3 treated with nusinersen

	SMA type 2a (*n* = 20)	SMA type 2b (*n* = 10)	SMA type 3a (*n* = 16)
Follow-up in months	34.5 (5–44)	36 (13–40)	33.5 (21–41)
Number of injections	11.5 (4–14)	11.5 (6–15)	11 (8–13)
			
HFMSE score	16 (0–41)	20 (4–51)	55.5 (31–64)
△HFMSE score	5 (−3–24)	4 (−2–13)	6 (−3–15)
WHO motor milestone score	1 (0–4)	1 (1–5)	6 (2–6)
Use of ventilator support ***n*** (%)			
None	15 (75)	10 (100)	16 (100)
Non-invasive ventilation	5 (25)	0 (0)	0 (0)
Hours of non-invasive ventilation	12 (12)	n.a.	n.a.
Invasive ventilation	0 (0)	0 (0)	0(0)
Dependent on feeding tube ***n*** (%)			
None	16 (80)	7 (70)	16 (100)
Nasogastric tube	0 (0)	1 (10)	0 (0)
Gastrostomy	4 (20)	2 (20)	0 (0)

SMA = spinal muscular atrophy; *n* = number; HFMSE = Hammersmith Functional Motor Scale Expanded; △HFMSE = difference in HFMSE score between baseline and last follow-up assessment, WHO = World Health Organization.

Outcomes present the data of last follow-up assessment, unless otherwise stated.

All outcomes are given in median (range) unless otherwise stated.

HFMSE scores increased in a non-linear fashion (*P* < 0.001) with a mean increase of 1.8 (CI 1.2–2.4) points after 6 months, 3.1 (CI 1.9–4.3) points after 12 months, 3.9 (CI 2.3–5.6) points after 18 months, 4.3 (CI 2.2–6.5) points after 24 months and 4.1 (CI 1.6–6.9) after 30 months of treatment, all in comparison to baseline scores (Fig. 1C and 2C, Table 3). After 12 months, motor function reached a relative plateau. Shorter disease duration at treatment initiation was associated with better treatment response (*P* < 0.001). Sixteen children (57%) showed an improvement of ≥3 points on the HFMSE. Seven children (25%) showed a decline of motor function during treatment compared with baseline, with a maximum decline of 3 points on the HFMSE. Six patients lost 1 or 2 points on the HFMSE, either because of increasing severity of contractures (*n* = 2) or inability to perform one specific item on the HFMSE (*n* = 5). One child lost 3 points after scoliosis surgery. Motor function in all seven children remained stable afterwards.

### Motor function assessments and milestones in SMA type 3

Twelve out of 16 children (75%) with SMA type 3a were ambulant at start of treatment, one was able to stand without support, and three were able to stand with help. One child regained the ability to stand alone after seven injections, but lost this milestone 4 months later.

Increases in HFMSE scores were non-linear (*P* < 0.001), with a mean increase of 1.8 (CI 1.2–2.5) points after 6 months, 3.2 (CI 1.9–4.5) points after 12 months, 4.1 (CI 2.4–5.9) points after 18 months, 4.6 (CI 2.2–6.9) points after 24 months and 4.6 CI (1.7–7.5) points after 30 months of treatment, all in comparison to baseline scores (Fig. 1D and 2D, Table 3). After 24 months, motor function reached a relative plateau. Shorter disease duration at treatment initiation was associated with better treatment response (*P* < 0.001). Ten (63%) children showed an improvement of ≥3 HFMSE points. Four patients (25%) showed a decline of motor function, losing up to 3 HFMSE points. All four children lost points on specific items due to increasing hip flexor contractures, but stabilized in the consecutive assessments.

### Natural history of SMA compared with disease course with treatment

**Figure 3:**
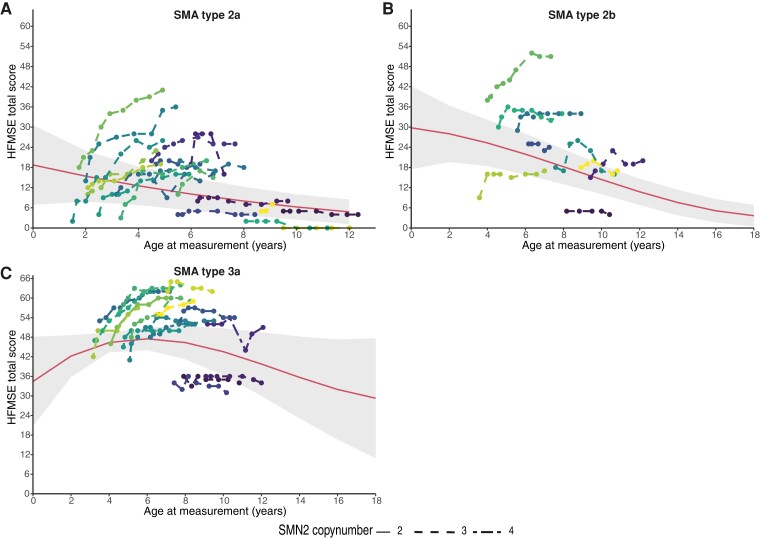
**Disease course with nusinersen treatment and natural disease course in children with SMA type 2a, 2b and 3a.** Individual motor function trajectories of symptomatic children treated with nusinersen (*n* = 42) are represented as individual (coloured) lines. Each line represents an individual child treated with nusinersen. The individual motor function scores of children treated with nusinersen improved or remained stable (as shown in Fig. 2) in 42 out of 44 (95%). This reflects a change from the natural history of SMA that is reflected by the red line with the 95% confidence intervals in grey. This natural history trajectory was reconstructed from 44 age group-matched, treatment-naive Dutch SMA patients with SMA types 2a (*n* = 16), 2b (*n* = 12) and 3 (*n* = 16) included in the Dutch natural history cohort. We analysed their motor function scores using a linear mixed-effects model with age, SMA type and an interaction term of these two predictors as fixed factors, whilst dependency in the data due to repeated measures was accounted for by a random intercept per individual, as previously published (2). As a result, this natural disease course trajectory shows that a child with SMA type 2a of 4 years old has a mean HFMSE score of 12 points, while at age of six the score will have deteriorated to approximately 10 points. (**A**) Individual trajectories of the HFMSE scores of 19 children with SMA type 2a, treated with nusinersen. The median increase of the HMFSE score during treatment was 5 points. Twelve (63%) children showed improved motor function, six (31%) remained stable and one (5%) showed a decline of 3 points during treatment. (**B**) Coloured lines are individual HFMSE score trajectories of nine children with SMA type 2b, treated with nusinersen. The median increase of the HMFSE during treatment was 4 points. Five children (55%) showed improved motor function and four (45%) remained stable during treatment. (**C**) Coloured lines are individual HFMSE score trajectories of 16 children with SMA type 3a, treated with nusinersen. The median increase of HFMSE scores during treatment was 6 points. Eleven (68%) children showed improved motor scores, four (25%) remained stable and one (6%) showed a decline in motor function. For a more detailed motor function trajectory of the natural history cohort (see Supplementary Figure 1).

We compared the disease course of children treated with nusinersen to data from our natural history cohort (23,5). We included all children from the natural history cohort with available longitudinal follow-up data available (28 with SMA type 2, 16 with SMA type 3). Median age at baseline was 6.2 years (range 1.4–17.2 years). Median follow-up was 45 months (range 3–90) in SMA type 2 and 38 months (range 3–74) in SMA type 3a. Disease course during treatment with nusinersen differed from natural history (Fig. 3, Supplementary Figure 1). We additionally compared the disease course of 15 children before and after start of treatment (3 SMA type 2a, 4 type 2b and 8 type 3a) with a median pre-treatment follow-up of 14 months (range 1–68). One patient with SMA type 2a, with a stable HFMSE score prior to treatment, showed an increase of 1 point after the start of nusinersen. Two children with type 2b with decreasing scores prior to treatment showed an increase in the HFMSE score after the start of nusinersen: one (67 months of age at baseline) had an increase of 5 points after six injections, the other (94 months of age at baseline) had an increase of 8 points after six injections. Two children with SMA type 3a with decreasing HFSME scores prior to treatment also improved after six injections: one 10 points (61 months of age at baseline), the other 4 points (92 months of age at start of treatment), respectively.

### Bulbar and respiratory function during treatment

Ten children with SMA type 1 did not receive mechanical ventilation at treatment initiation. Three of them (1 type 1b and 2 type 1c) started non-invasive mechanical ventilation (NIV) during treatment (age 2 months for type 1b and 11 and 46 months for type 1c at treatment initiation, respectively), despite improvements of motor function in all three. Two out of 4 children with SMA type 1c who received invasive ventilation prior to treatment, reduced their ventilation time from 22h/d to 13 and 12h/d, respectively (Table 2). Three children (2 type 1b and 1 type 1c) had to start tube feeding during treatment, all after six injections.

One child with SMA type 2 (type 2a) started nocturnal NIV (8 h per night) after four injections at the age of 9.5 years. One child with SMA type 2b required nasogastric tube feeding after 12 injections to prepare the child for scoliosis surgery (Table 3); two children with type 2a did no longer need tube feeding after seven and eight injections, respectively.

None of the children with SMA type 3a required tube feeding or mechanical ventilation prior to treatment, which did not change during treatment.

### Motor function and milestones after start of treatment in two presymptomatic patients

Two infants were diagnosed before onset of clinical symptoms and were therefore classified as ‘presymptomatic’.

The first infant (two *SMN2* copies) was diagnosed prenatally because of an older sibling who died of SMA type 1b. The infant showed no signs of SMA after birth and had a baseline CHOP-INTEND score of 43 points. The infant started treatment at Day 4. The infant was able to sit independently at the age of 11 months, to stand with support at 19 months and walk with support at 30 months. He needed NIV (13 hours/day) and nasogastric tube feeding from the age of 18 months.

The second child (three *SMN2* copies) was diagnosed at the age of 3 months after a recent diagnosis of SMA type 3 in an older sibling. This presymptomatic infant showed no clinical signs of SMA and had a baseline CHOP-INTEND score of 62 points. The infant started treatment at day 102. He was able to walk with support at the age of 10 months and to walk without support at the age of 21 months. Respiratory and bulbar function remained normal until last follow-up (34 months).

## Safety

We observed 82 treatment-related adverse events in 45 patients (63%) on a total of 934 injections (9%) (Table 4). The most reported side effects were headache, back pain, pyrexia and discomfort. One patient developed aseptic meningitis one day after his first injection. He fully recovered and continued treatment with nusinersen without further complications. One child had an allergic skin reaction with angio-edema of the lips shortly after the second and fourth injection. Skin allergy tests with nusinersen and drugs used for anaesthesia were normal. Despite the use of clemastine, the skin rash occurred twice more without angio-edema. Two children showed an increased intracranial pressure measured prior to their injection of nusinersen. Both children were without concomitant clinical symptoms. Investigation with MRI and fundoscopy showed no signs of hydrocephalus. Both children continued treatment with nusinersen.

**Table 4:** Reported adverse events during treatment with nusinersen

Adverse event	Number of reported AE (*n* of patients)
(Allergic) skin reaction	5 (3)
Aseptic meningitis	1 (1)
Increased intracranial pressure (without hydrocephalus)	3 (2)
Headache	24 (20)
Back pain	10 (9)
Tingling feeling in back/leg	4 (4)
Discomfort	13 (6)
Pyrexia	13 (10)
Proteinuria	9 (8)

Number of reported AEs in 934 injections.

AE = adverse event; *n* = number.

## Discussion

This study is a population-based assessment of efficacy of intrathecal treatment with nusinersen in Dutch children with SMA types 1–3a. We observed improvement of motor function scores in 72% of patients with SMA types 1b-3a and 23% of the patients gained new motor milestones. In another 18% of the patients, motor function scores stabilized during treatment. This is a clear difference from the expected natural history patterns, observed in 90% of young symptomatic children. Importantly, we did not observe a consistent decline of motor function in any of the enrolled patients. Longitudinal analysis showed that motor function changes after the start of treatment are non-linear, with the most pronounced improvement within the first 12 months of treatment, followed by a relative stabilization of motor function after 30 months. In contrast, bulbar and respiratory function did not improve during treatment. Shorter disease duration at time of treatment initiation was associated with a better treatment response. The national single centre treatment setting minimizes the risk of inclusion bias and reduces variability in outcome measure assessments. Our data therefore provide an important insight in real-world treatment efficacy of nusinersen for children with SMA.

Response to treatment as previously defined as improvements of ≥4 CHOP-INTEND or ≥3 HMFSE points,^10,11,35^ was observed in 85% of children with SMA type 1, 57% of those with type 2 and 63% of those with type 3.

Nine children (39%) with SMA type 1 learned to sit independently during treatment. The percentage of children that achieved this motor milestone is higher than in the randomized controlled ENDEAR trial and other reports^10,26,6–8^ and may reflect differences in baseline characteristics, in particular disease duration and condition at the start of treatment. The median CHOP-INTEND baseline scores of children with SMA type 1 who learned to sit was 32 (range 20–46) which is in line with previous findings.^26^ Parents of babies with SMA type 1b in already poor condition often opted for best supportive care. This explains the underrepresentation of this group of patients in our treatment cohort.

The percentage of children with SMA types 2 and 3 improving ≥3 HMFSE points in our cohort is in line with the results of the randomized CHERISH study. However, the responder rate in children with SMA type 2 in our cohort is higher in comparison to another study which had a shorter follow-up time (i.e. 12 months).^25^ The extended follow-up time of our study revealed that treatment response is reflected by incremental improvements up till 30 months of treatment followed by stabilization of motor function scores.

An increase of ≥1 on specific items or, an increase in the score for kicking of ≥2 points on the HINE-2, > 4 points on the CHOP-INTEND or ≥3 points on the HFMSE were previously defined as evidence for treatment efficacy in the sham-controlled clinical trials with nusinersen, because this is uncommon in the natural course of disease.^10,11,35^

Various motor scales and assessments are validated and used to follow up on SMA patients. Still there is no consensus on what changes are clinically significant or meaningful.^39,40^ However, any improvement of functional motor scales above the age of 5 years or even stabilization is change from the natural course of SMA and should therefore be interpreted as a treatment effect.^41^ This is further illustrated by the comparison of the historical cohort of children enrolled in the Dutch natural history study.

We observed temporary functional decline under treatment most often in children with progressive contractures or scoliosis. This underscores that pro-active treatment of contractures is essential to maintain and optimize motor function in children who are treated with new therapies for SMA.^42,43^

The pattern of treatment response—improvement followed by a plateau—was similar in all SMA types, but the time-to-plateau differed. We assume that this pattern is best explained by accelerated maturation or activation of ‘hibernating’ motor units,^44^ while the plateau phase reflects the limitation in the number of motor units that can be revived. We cannot exclude that the longer time to plateau in SMA type 1 is explained by the longer treatment time needed for motor neurons to recover. However, properties and limitations of motor scales may provide an alternative explanation, including ceiling effects and the non-continuous nature of motor function scales that may mask subtle improvements, in particular in non-ambulant children with SMA types 2 and 3a.

The percentage of treatment responders differed between SMA types. This is probably due to the selection of patients with SMA type 1 (as outlined above) and the relatively long disease duration of children with SMA type 2 at the start of treatment. Disease duration was the most important predictor of the magnitude of treatment effect. Children with SMA type 2 starting after the age of 5.5 years and type 3a starting after 7.5 years showed limited or no improvement. However, we observed clinical stabilization in these patients, which is an important change from the natural disease course that likely reflects treatment effects. The importance of early treatment and its effect on motor response was shown in our data from symptomatic patients with SMA types 1–3 and underlined by the motor performance of our two presymptomatically diagnosed and treated infants. Newborn screening programs would make presymptomatic treatment the rule rather than the exception and should be a priority in all countries with reimbursement arrangement for genetic therapies.^45,46^

Combinatorial therapies that would target SMN and other, non-SMN targets that support the neuromuscular system (i.e. pyridostigmine for neuromuscular junction (NMJ) function, and myostatin inhibitors currently in clinical trials to support muscle function) might have a synergistic effect on motor function and daily life performance. Combining multiple SMN-targeting therapies remains controversial as it is unclear if there are limits to up-regulating SMN levels (see e.g.^47^).

Treatment effects of nusinersen on respiratory and bulbar function are limited and clearly fall behind the observed motor function changes. We previously reported the lack of persistent improvement of bulbar function in children with SMA type 1 treated with nusinersen.^48^ Although the hours of mechanical (non-)invasive ventilation declined on average during follow-up in a cohort of 123 French children with SMA type 1, effects on respiratory function were clearly less pronounced than those on motor function.^16^ Other studies also report limited effects of nusinersen on respiratory and bulbar function^49–57^. We compared the hours of ventilation or the need of tube feeding before and after the start of treatment. We acknowledge that this approach to assess respiratory and bulbar function may lack sensitivity to detect subtle, but relevant changes. Parents frequently reported changes, including a louder voice or cry, a decreased mealtime or improving intake and fewer respiratory infections during nusinersen treatment. Patient reported outcome measures might therefore provide valuable insights but need to be standardized and validated for future use.

The overall occurrence of adverse events in our cohorts was low and the procedure well tolerated. None of the children discontinued treatment because of adverse events. Anaesthesia and sedation were well tolerated. Intrathecal injections could be performed without complications in 45 patients with either (severe) scoliosis or previous spinal surgery, without the use of radiologic assistance, e.g. CT-, fluoroscopy- or radioguidance^58–62^.

This study has several important strengths. First, this is a single-centre population-based SMA cohort. We systematically obtained motor function data prior to the start of and during treatment, which minimizes inclusion bias and contributes to the real-world evidence for nusinersen efficacy. We applied longitudinal analyses and showed a non-linear pattern of improvement. This allowed us not only to show improvement after the start of treatment, but also and for the first time, stabilization in children who had longer disease duration. The comparison of treatment effects with natural history data in patients with the same supportive care strategy (e.g. same country and treating physicians) has not been reported before. Limitations of our work include the sample size and the selection bias of children with SMA type 1b.

We conclude that intrathecal nusinersen treatment of patients with SMA is safe and results in modest to large effects in the large majority of young patients with SMA types 1, 2 and 3a. The effect of nusinersen treatment is most evident in in the first year of treatment. Short disease duration at treatment initiation is related to a larger treatment effect. Uncertainties regarding the longer-term duration of these effects and effects on respiratory and bulbar function remain to be resolved in future studies.

## Supplementary Material

fcac269_Supplementary_Data

## Abbreviations

CHOP-INTEND =: Children's Hospital of Philadelphia Infant Test of Neuromuscular Disorders
CI =: 95% confidence interval
HINE-2 =: Hammersmith Infant Neurological Examination-2
HFMSE =: Hammersmith Functional Motor Scale Expanded
LMM =: linear mixed models
NMJ =: neuromuscular junction
SMA =: spinal muscular atrophy
SMN =: survival motor neuron.
