# Supplementary material for: Population-based assessment of nusinersen efficacy in children with spinal muscular atrophy: a 3-year follow-up study
Source: Brain Commun. 2022 Oct 31;4(6):fcac269. doi: 10.1093/braincomms/fcac269 (PMC9651026; doi:10.1093/braincomms/fcac269)
Supplement: fcac269_Supplementary_Data [file fcac269_supplementary_data.docx]

**Supplementary FIGURE 1: Individual data of treated (with nusinersen) and non-treated patients with SMA**

**Legend Supplementary Figure 1:**  A) Motor function trajectories of children with SMA type 2a without treatment (purple, n=16) and with nusinersen treatment (yellow, n=20). Showing a different disease trajectory between both groups: children with treatment show an increase of motor function where patients without treatment show a clear decline of motor function. B) Motor function trajectories of children with SMA type 2b without treatment (purple, n=12) and with nusinersen treatment (yellow, n=10). Showing a different disease trajectory between both groups: children with treatment show an increase followed by stabilization of motorfunction, where patients without treatment show a clear decline of motorfunction. C) Motor function trajectories of children with SMA type 3a without treatment (purple, n=16) and with nusinersen treatment (yellow, n=16). Showing a different disease trajectory between both groups: most children with treatment show an increase of motorfunction followed by a stabilization, where patients without treatment show a lower maximal motorfunction score (if there was an increase compared to baseline) followed by a decline of motorfunction. Children with a lower baseline HFMSE show stabilisation, while patients with the same age without treatment show a clear decline of motorfunction, which supports the idea that treatment with nusinersen at least gives stabilization of motorfunction.
